# Anxiety symptoms and associated functional impairment in children with CHD in a neurodevelopmental follow-up clinic

**DOI:** 10.1017/S1047951122001767

**Published:** 2022-06-20

**Authors:** Sean Cunningham, Kathleen Campbell, Thomas Miller, Sarah Winter, Angela Presson, Zhining Ou, Kristi Glotzbach

**Affiliations:** 1 Department of Pediatrics, Division of General Pediatrics, University of Utah, Salt Lake City, UT, USA;; 2 Department of Pediatrics, Division of Developmental and Behavioral Pediatrics, The Children’s Hospital of Philadelphia, Philadelphia, PA, USA;; 3 Department of Cardiology, Pediatrics, Maine Medical Center, Portland, ME, USA;; 4 Department of Internal Medicine, Division of Epidemiology, University of Utah, Salt Lake City, UT, USA; 5 Department of Pediatrics, Division of Critical Care Medicine, University of Utah, Salt Lake City, UT, USA

**Keywords:** Congenital health disease, anxiety, children, cohort study

## Abstract

**Objectives:**

To examine the prevalence of anxiety symptoms and associated functional impairment to adaptive skills among elementary-aged children with CHD and to determine the need for anxiety screening in this high-risk population.

**Study design:**

In a single-centre retrospective, cohort design, caregivers reported anxiety symptoms using *Conner’s* scales and functional impairment to adaptive skills using the *Adaptive Behavior Assessment System*. A total of 194 children were stratified across two cohorts: early elementary (ages 3–6 years) and late elementary (ages 6–14 years). Descriptive statistics summarised the frequency of anxiety symptoms and functional impairment. Spearman’s correlations compared anxiety symptoms to functional impairment of adaptive functioning. Univariable logistic regressions examined demographic and clinical characteristics associated with anxiety symptoms.

**Results:**

The majority of patients presented with anxiety, early elementary (63%), and late elementary cohorts (78%). Functional impairment was moderately correlated with anxiety symptoms in the early elementary cohort (*r*_s_ = −.42, 95% CI [−0.58, −0.21], p = <.001). Greater anxiety symptoms were associated with lower cardiac complexity at primary age of surgery in the late elementary cohort (OR = 12.15, p = 0.019). Lesser anxiety symptoms were associated with having private insurance (OR = 0.25, p = 0.014).

**Conclusion:**

This study demonstrates anxiety symptoms are common and associated with functional impairment to adaptive functioning in younger children with CHD. No clear clinical predictors exist for anxiety symptoms or functional impairment; therefore, screening for anxiety symptoms may need to be added to standard clinical assessment of all children with CHD participating in neurodevelopmental follow-up.

CHD is the most common birth defect, making up 1% of all live births per year. One in four children with CHD will require surgery in their first year of life.^1–3^ Anxiety symptoms are common in children with chronic medical conditions, including children with CHD. The prevalence of anxiety disorders has been found to range from 5 to 50% in children with chronic medical conditions such as asthma, epilepsy, and diabetes^4^ and 26 to 30% in children with CHD.^5–7^ Increased anxiety symptoms and diagnoses in these populations compared to the general population may be related to childhood medical trauma as a result of separation from parents, long and frequent hospitalisations, exposure to painful procedures shortly after birth, and potential modifiable aspects of parental mental health (e.g., anxiety).^8,9^

The consequences of anxiety in childhood are significant, have long-term effects on individuals’ lives, and contribute to preventable hospital and societal costs. Anxiety may exacerbate the course of the child’s medical illness and correlate with increased psychiatric and physical comorbidities in children.^10,11^ Children with anxiety disorders have increased healthcare utilisation, school absences, and poorer disease outcomes compared to those without anxiety.^9^ Childhood anxiety is also associated with social and behavioural problems, poor academic performance, depression, substance use, attention-deficit/hyperactivity disorder, conduct disorder, suicidal behaviour and even death by suicide.^9^ Left undiagnosed and untreated, anxiety also increases risk for dysfunction in adulthood and is linked to significant psychiatric disorders and substance abuse.^10,12^

Anxiety symptoms are common in young children with CHD. A recent, single-centre study that examined a large population of children between the ages of 4 and 17 years old with and without CHD presenting for acute care (n = 118,785) found ~16% of the children with CHD aged 4 to 9 years old had a diagnosis or utilised a medication for anxiety and/or depression.^13^ Other studies have found the presence of anxiety symptoms in older children, adolescents, and adults with CHD in 26–30% of patients.^5–7,14^ Thus, at younger ages and throughout the lifespan, anxiety and anxiety symptoms have been found in individuals with CHD with associated deleterious outcomes.

In 2012, the American Academy of Pediatrics and the American Heart Association released a scientific statement providing an assessment structure for the longitudinal neurodevelopmental evaluation and care for high-risk children with CHD.^15^ International consortiums and working groups have subsequently outlined specific batteries of standardised and validated measures intended to capture the neurodevelopmental vulnerabilities in the clinical care and treatment of children with CHD.^16,17^ Broad-based behavioural measures are recommended; however, targeted anxiety symptom assessments are not a suggested component of core assessment batteries during routine neurodevelopmental followup. Anxiety assessments are suggested starting for children ages 6 years old and reaching into young adulthood; however, younger children have the potential for deleterious anxiety, but screening is not recommended for this age group. Given the presence of anxiety in younger-aged children (i.e., 4 years), anxiety symptom screening may be necessary, yet missing, as a targeted area of assessment across a wider age spectrum to capture the full age range of children who may be experiencing anxiety in routine neurodevelopmental follow-up visits.

Children with CHD are at a greater risk for neurodevelopmental challenges and psychological disorders. Neurodevelopmental follow-up programmes are designed to provide screening for psychological disorders and can be a part of clinical care where anxiety conditions are identified in children with CHD. Given the effects of anxiety in children with CHD on the individual and society, early and proper identification and treatment of anxiety disorders has been proposed in the literature.^11^ Information on the prevalence of anxiety symptoms identified in neurodevelopmental follow-up programmes may help guide research priorities, clinical care, and allocation of healthcare resources. Therefore, it is important to know how common symptoms of anxiety are in children with CHD routinely screened for psychological conditions, such as in neurodevelopmental follow-up programmes.

Children with CHD are at greater risk for neurodevelopmental challenges (e.g., neuropsychiatric functioning) when compared to the general population. Thus, it is important to know how common anxiety and associated functional impairment to adaptive skills are across the age spectrum of children with CHD. There may be ways to improve the current system of evaluating anxiety in a population of children with anxiety symptoms, including patients who may be going underidentified for early and appropriate treatment of anxiety-related symptoms in neurodevelopmental follow-up programmes. The current literature lacks systematic exploration of younger children under routine follow-up neurodevelopmental care specifically for anxiety symptoms. Additionally, current studies have not examined associated functional impairments related to anxiety. Examining younger children specifically for anxiety and associated functional impairments can assist in early identification and treatment efforts. Given the paucity of studies that examine anxiety symptoms across a wide age spectrum of children with CHD, and associated functional impairment, we aimed to study elementary-aged children under routine care in a large, neurodevelopmental follow-up programme to determine if anxiety symptoms are present across the elementary age spectrum and how those symptoms correlate with functional impairments. Although there are several ways that children can be followed up for care, our centre utilises an interdisciplinary neurodevelopmental follow-up programme to assess for neuropsychiatric and developmental challenges among children with CHD.

## Materials and methods

### Study and patients

Our design is a retrospective, single-centre, cohort study of children aged 3–14 with CHD who underwent neuropsychological evaluations between July 2015 and June 2020. All patients met criteria for longitudinal cardiac neurodevelopmental follow-up programme based on the American Heart Association guidelines for high-risk CHD.^15^ Patients were included if they had CHD and underwent catheter based or surgical intervention. Patients were screened for anxiety symptoms as part of an outpatient, interdisciplinary neurodevelopmental follow-up clinic visit. Each interdisciplinary clinic visit is staffed by a developmental paediatrician, cardiologist, psychologist, speech and language pathologist, nurse, and physical therapist who perform assessments in their respective fields. Children were stratified into two groups contingent on age and neuropsychiatric measure. Children aged 3–6 years were classified in the early elementary cohort, and children aged 6–14 were in the late elementary cohort. Three children aged 6 years old from the early elementary cohort were given the measure for the older cohort and were included in late elementary cohort for analysis. The study was approved by the University of Utah Institutional Review Board and was exempt from patient and parental consent.

### Demographic and clinical variables

Demographic and clinical variables were collected from the electronic medical record.^18^ Cardiac anatomy at the time of the index operation was grouped into two categories: (1) single ventricle and (2) two ventricle. Risk Adjustment for Congenital Heart Surgery-1 (RACHS-1) scoring system was used to define the index surgical complexity [low complexity = 1–2; medium complexity = 3–4; high complexity = 5–6].

Critical CHD was defined as requiring surgery in the first year of life. Post-operative complications (intraventricular haemorrhage, stroke, seizure, arrhythmia, extracorporeal membrane oxygenation, necrotising enterocolitis, pacemaker placement, thrombosis, and systemic ventricular dysfunction) were combined to create a dichotomous variable and categorised as yes if the child experienced one or more complication during their index hospital stay. A dichotomisation of cardiac complications was necessary as the frequency of complications did not allow for group comparison. Insurance coverage was categorised as commercial (private), Medicaid (public), or self-pay. Annual household income as a surrogate for socioeconomic status was approximated with median household income by county/zip code and treated as a continuous variable for analysis.^19^

### Measures of anxiety symptoms

The *Conners Early Childhood* is a norm-referenced, caregiver-rated measure of behavioural, emotional, social, and developmental milestones in children aged 2 to 6 years old.^20^ The *Conners Early Childhood* identifies anxious symptoms using a *T*-score system.^20,21^ Anxiety symptoms are defined at a T-score on the *Anxiety* scale at T ≥ 60, equal to or greater than one standard deviation above the population mean (i.e., M = 50; SD = 10).^20^ *Conners Early Childhood* T-scores below 60 indicate a “low score” or “average score” and were not considered consistent with anxiety symptoms.

The *Conners Comprehensive Behavior Rating Scales* is a normreferenced caregiver-rated measure of childhood disorders based on the Diagnostic and Statistical Manual of Mental Disorders (DSM-IV-TR; American Psychiatric Association, 2000).^22^ The *Conners Comprehensive Behavior Rating Scales* assesses children aged 6 to 18 years old. The *Conners Comprehensive Behavior Rating Scales* examined the presence of anxiety symptoms as defined by Diagnostic and Statistical Manual of Mental Disorders criteria; each manifestation relates to distinct anxiety diagnoses including generalised anxiety disorder, separation anxiety disorder, social phobia, and obsessive-compulsive disorder. The authors of the *Conners Comprehensive Behavior Rating Scales* offer the same interpretation of anxiety symptom presence, whereas measurement is based on T-score and is defined by the same parameters as the *Conners Early Childhood*.^22^ Thus, as with the *Conners Early Childhood*, the presence of anxiety symptoms on the *Conners Comprehensive Behavior Rating Scales* and Diagnostic and Statistical Manual of Mental Disorders Scales was defined by *T*-scores, *T* ≥ 60, equal to or greater than one standard deviation above the population mean; scores below 60 indicate a “low score” or “average score” and were not considered consistent with anxiety symptoms.^22^ The *Conners Comprehensive Behavior Rating Scales* have an internal consistency ranging from .69 to .96.

### Measure of functional impairment

The *Adaptive Behavior Assessment System, Third Edition* measures functional impairment to adaptive skills by obtaining caregiver ratings across a number of different developmental skill areas quantified into an overall Global Composite Scale.^23^ The Global Composite Scale is comprised from separate composite scores that each have a specific focal area of development, conceptual composite (i.e., communication, academics, self-direction), social composite (i.e., leisure, social skills), and a practical composite (i.e., community use, home living, health and safety, self-care). Functional impairment on the global composite scale was defined by a standard score of ≤ 85, one standard deviation or greater below the population mean for each of the global and composite scales.

### Statistical analysis

Age and hospital length of stay were summarised by using median and interquartile range due to distribution skew. Next, counts and percentages for categorical variables were reported. Univariate logistic regression models were computed for each cohort to examine the association between demographic and clinical variables and anxiety. Since the Risk Adjustment for Congenital Heart Surgery had few patients in the low complexity group, Firth’s penalised logistic regression was used to reduce bias. Odds ratios and 95% confidence intervals were reported. The value of correlation ranges between −1 and 1, where 0 means no monotonic relationship. The ranges of 0.0–0.1, 0.1–0.4, 0.4–0.7, 0.7–0.9, 0.9–1.0 correspond to the strength of relationship being negligible, weak, moderate, strong and very strong as a rule-of-thumb.^24^ The monotonic pairwise evaluated associations between anxiety domains and functional composites using Spearman’s rank correlations within each cohort. Correlation coefficients (r_s_), their 95% confidence intervals, and their asymptotic *p*-values are used to report relationships between variables of anxiety and functional adaptive skills. Statistical significance was assessed at the 0.05 level. Statistical analyses were implemented using R v. 4.0.3.^25^

## Results

Demographic and clinical characteristics for the sample are outlined in Table 1. Our total sample consisted of 194 participants, 78 children in the early elementary cohort and 116 in the late elementary cohort. The median age was 4.8 years (interquartile range 4.3, 6.0) in the early elementary cohort and 9.3 years (interquartile range 8.2, 11.8) in the late elementary cohort. In both groups, participants were mostly male, Caucasian, had private insurance, and were RACHS-3 and 4 surgical cases. The majority of patients had two ventricle physiology, 69% in the early elementary group and 53% in the late elementary group. Sixty-six per cent of the full cohort experienced at least one complication: intraventricular haemorrhage (0%), arrhythmia (41%), extracorporeal membrane oxygenation (2%), necrotising enterocolitis (10%), pacemaker (8%), stroke (8%), seizure (8%), thrombosis (17%), systolic dysfunction (23%), and infection (7%).

**Figure 1.**
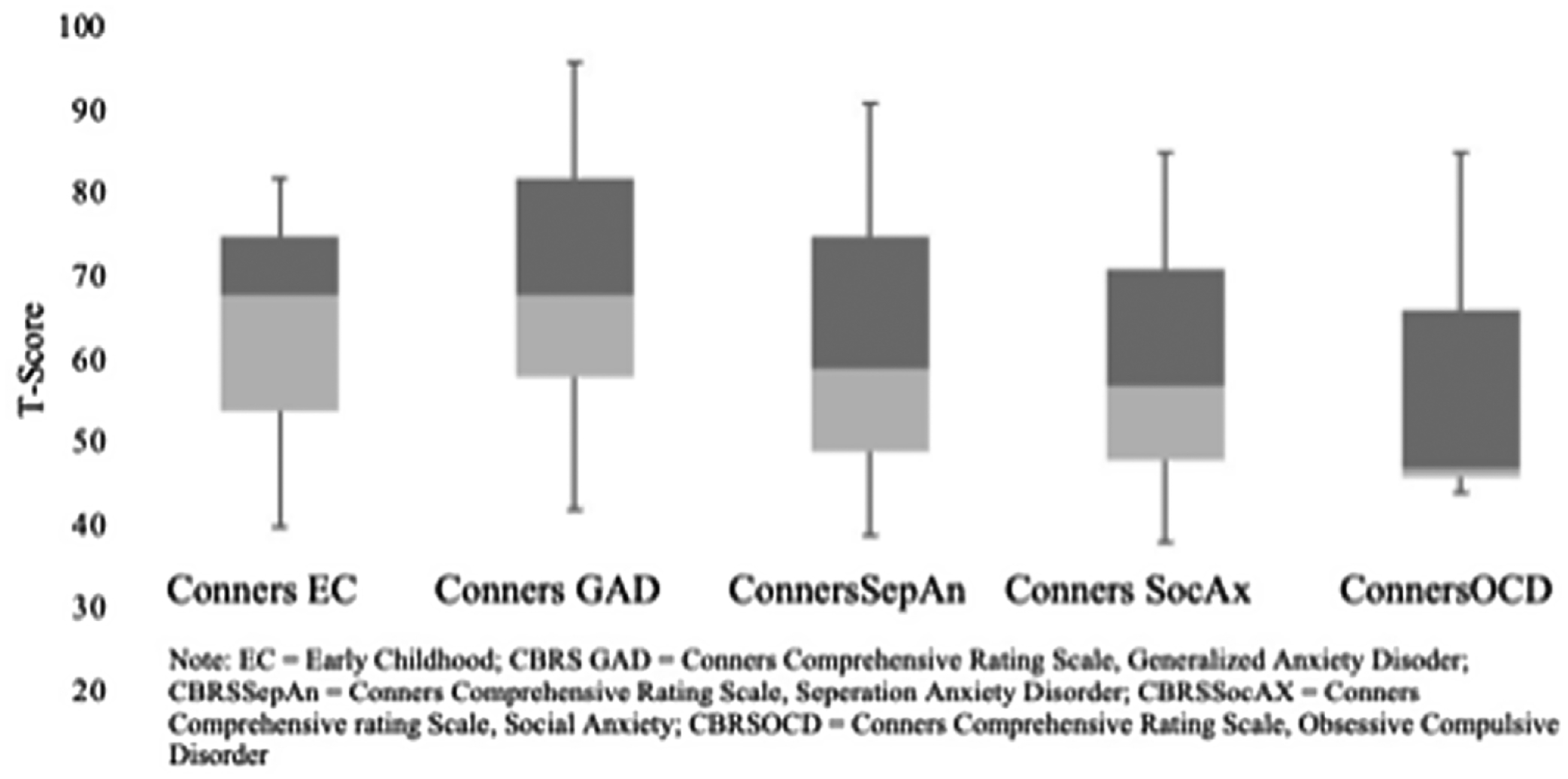
Prevalence of anxiety symptoms for early and late elementary cohorts.

In the early elementary cohort, 63% of children showed anxiety symptoms, whereas in the late elementary cohort 78% showed anxiety symptoms. The median anxiety T-score on the *Conners Early Childhood* was 67.5 (interquartile range 4.3, 6.0). In the late elementary cohort, median T-scores for the *DSM-IV* scales of the *Conners Comprehensive Behavior Rating Scales* were as follows: generalised anxiety disorder, median 69.3 (interquartile range 58.8, 82.0); separation anxiety disorder, median 59.0 (interquartile range 49.0, 75.0); social phobia, median 57.0 (interquartile range 48.0, 71.0); and obsessive-compulsive disorder, median 53.0 (interquartile range 46.0, 66.0). Figure 1 illustrates the score distribution of the anxiety scales across the early and late elementary cohorts.

**Figure 2.**
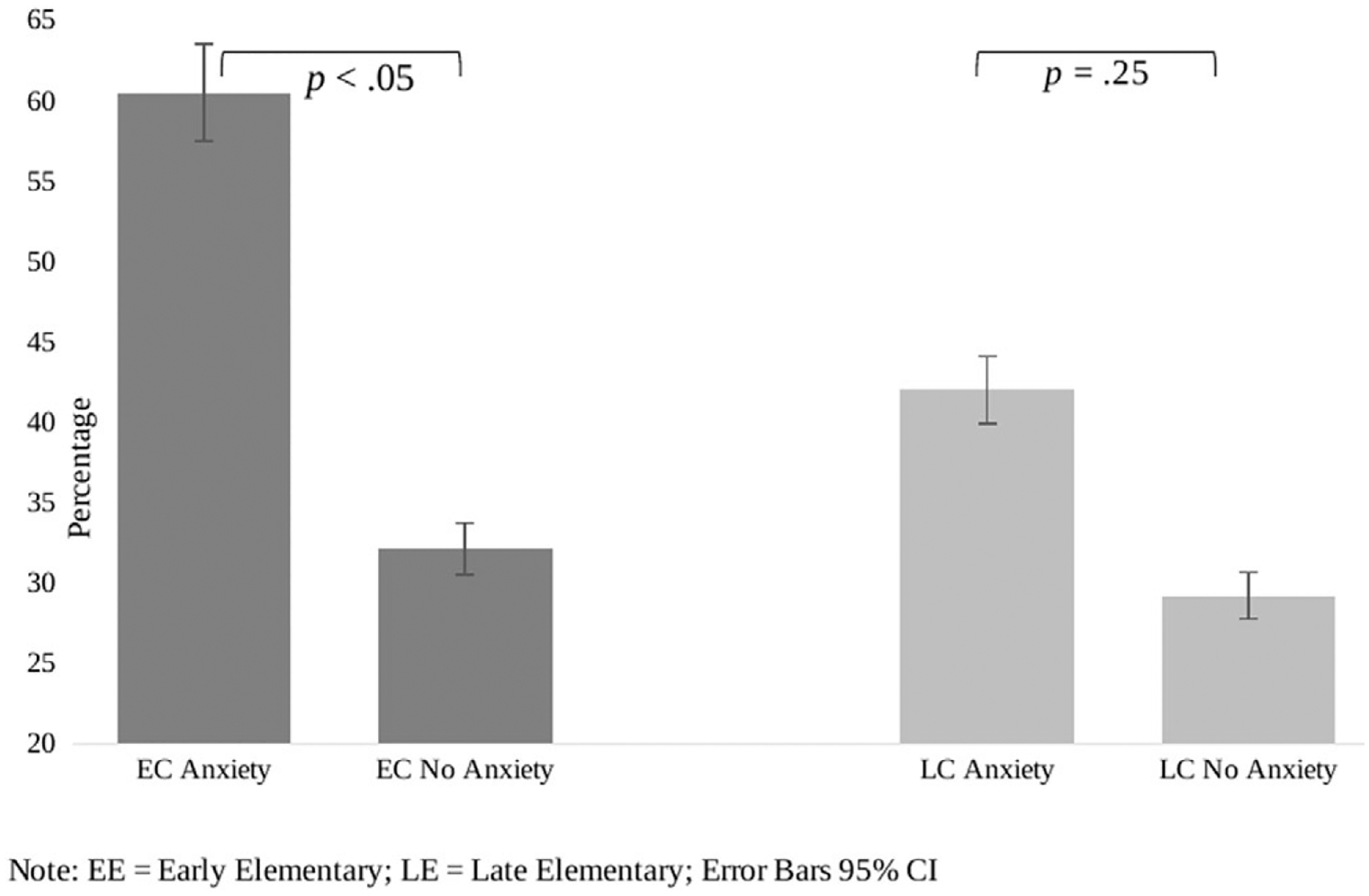
Percent of cohort with functional impairment.

Global functional impairment was present in 49% of children in the early elementary cohort (Adaptive Behavior Assessment System, Third Edition median = 86.0, interquartile range 75.5, 94.0) and 39% in the late elementary cohort (Adaptive Behavior Assessment System, Third Edition median = 90.0, interquartile range 80.8, 97.0). In the early elementary cohort, of children experiencing anxiety symptoms, 61% also had global functional impairment. In the late elementary cohort, of those children experiencing anxiety symptoms, 42% had associated functional impairment. There was a statistically significant difference in global functional impairment within groups. In the early elementary cohort, children with the presence of anxiety symptoms had more global functional impairment (61%) when compared to those who did not have anxiety symptoms (32%), p < .05. Figure 2 illustrates the percentage of functional impairments across the early and late elementary cohorts.

We utilised Spearman’s rank correlation coefficients and their 95% confidence intervals to examine functional composites and anxiety symptoms for the early elementary cohort and anxiety disorder domains for the late elementary cohort. In the early elementary cohort, anxiety scores were correlated with functional impairment (negatively correlated with scores of functioning) in all areas of adaptive functioning: moderate correlation with the general adaptive composite, r_s_ = −.42, 95% CI [−0.58, −0.21], p = <.001 and conceptual composite, r_s_ = −.41, 95% CI [−0.58, −0.21], p = <.001; weak correlation with the practical composite, r_s_ = −.30, 95% CI [−0.49, −0.09], p = <.001; and the social composite, r_s_ = −.28, 95% CI [−0.48, −0.05], p = <.001.

In the late elementary cohort, symptoms of generalised anxiety disorder were weakly or moderately correlated with all areas of functional impairment; general adaptive composite, r_s_ = −.26, 95% CI [−0.45, −0.05], p = .008; and the social composite, r_s_ = −.21, 95% CI [−0.38, −0.02], p = .034. Social anxiety disorder symptoms were weakly correlated with lower scores on the conceptual composite r_s_ = −.26, 95% CI [−0.46, −0.07], p = .007 and social skills composite r_s_ = −.24, 95% CI [−0.42, −0.04], p = .015. Obsessive-compulsive disorder and separation anxiety disorder symptoms were not correlated with lower level of functional impairment to adaptive skills.

In the univariable logistic regression, there was an association between anxiety symptoms and insurance type, with less anxiety symptoms in children having private insurance when compared to those with public insurance or self-pay in the early elementary cohort (OR = 0.25, 95% CI [0.10, 0.7], p = 0.014). This relationship was not present in the late elementary cohort. Lower cardiac complexity at primary surgery was associated with increased anxiety symptoms compared to high complexity in the late elementary cohort (OR = 12.15, 95% CI [1.4, 1598.89], p = 0.019. There were no associations between the presence of anxiety and any other demographic and clinical variables for the early elementary or late elementary cohorts (Table 2).

## Discussion

Our study examined the prevalence of anxiety symptoms and associated functional impairment to adaptive skills in elementary-aged children with CHD in a single-centre neurodevelopmental follow-up programme. We identified anxiety symptoms in 63% of our early elementary cohort and 78% in our late elementary CHD cohort. This prevalence exceeds the prevalence of anxiety symptoms in the general population and other medicalised populations.^4,26^ Anxiety symptoms correlated with functional impairment to adaptive skills in both the younger and older age cohorts. More specifically, we found associations between anxiety symptoms and a broader scope of functional impairments to adaptive skills in the younger elementary group, demonstrating more functional impairment in early elementary ages when anxiety is present, than when compared to the older elementary group. With regard to clinical and demographic variables, anxiety symptoms were associated with lower cardiac complexity at primary age of surgery in the later elementary cohort, and in the early elementary cohort, having private insurance. However, overall no clear clinical profile emerged to suggest risk of anxiety symptoms in any patient group in particular.

Our findings built upon on previous studies indicating there is an increased prevalence of anxiety symptoms in children with CHD^13^ and expanded to determine if targeted anxiety assessment would capture anxiety symptoms across the age spectrum, especially in younger ages, for purposes of preventative interventions. Our results demonstrate a high prevalence of anxiety symptoms with associated functional impairment in adaptive skills in a younger at risk patient population of children with CHD. That is, a greater association was demonstrated between the presence of anxiety symptoms and functional impairment in the younger-aged cohort studied, illustrating unique needs in this population for identification and intervention. Thus, without anxiety-specific screening targeted towards younger ages administered during routine neurodevelopmental evaluations that assess for neuropsychiatric outcomes, a context that can provide access to interventions, anxiety symptoms may not be identified. Additionally, consideration of the use of parent report survey tools to measure anxiety symptoms in young children with CHD can work to eliminate the threats to validity that may occur with self-report assessments at younger ages.^27^

When considering the impact of anxiety symptoms on functional impairment to adaptive functioning skills, it is important to study adaptive functioning across age groups. In the current study, the pathway from anxiety to functional impairment did not follow the same pattern among each cohort, implying separate aetiologies at play and an area of further investigation. More importantly, with more severe associations between anxiety and functional impairments in the younger-aged group, a greater need emerges to identify this population for treatment.

Anxiety symptoms were associated with lower cardiac surgical complexity, RACHS-1 category 1 and 2, in the late elementary cohort. This is discrepant from other studies and our assumption that higher surgical complexity would be associated with more significant comorbidities and thus anxiety symptoms. There is conflicting evidence in the literature about the association between anxiety and complexity of cardiac defects.^6,28–30^ There was inconsistency in our findings as they relate to clinical and demographic variables and associations to anxiety symptoms and previous studies on anxiety in children with CHD. In our study, the discrepancy between surgical complexity and anxiety symptoms may be related to the limitations of the RACHS-1 score. For example, 53% of children in the late elementary cohort had single ventricle heart disease but a larger proportion of patients in the low or medium complexity surgical group. This reflects the fact that the RACHS-1 categorisation system includes procedures commonly used for single ventricle palliation (i.e., aortic to pulmonary shunts, cavopulmonary anastomoses, and pulmonary artery banding) in the RACHS-1 category 2 and 3. Newer, validated surgical risk classification systems may be more effective to ameliorate these discrepancies in future studies. The lack of a clear clinical profile for anxiety symptoms and lower surgical complexity might also be related to programmatic and provider referral bias. Many of our youngest patients are automatically referred at the time of their principal surgical discharge and identified at high risk for neurodevelopmental impairment based on the American Heart Association criterion for evaluation. Older patients are more often seen in our clinic due to specific physician or parental concern for developmental delay, behaviour challenges, or mental health impairment. Additionally, this may also represent an era effect that as our programme started, we were seeing older patients by physician referral for neurodevelopmental concerns. Our group previously demonstrated this referral bias and clear benefit of neurodevelopmental evaluation regardless of anatomic lesion or surgical complexity.^31^ This suggests that in the absence of a clinical phenotype that predicts anxiety symptoms, all patients regardless of age, lesion or surgical complexity should be screened during routine follow-up visits.

There was a higher frequency of anxiety symptoms in the late elementary group. This may be due to greater awareness of medical procedures and risk to life. For example, previous studies have shown that children and adults with CHD specifically have elevated medical fears.^29,32^ In a study of post-traumatic stress disorder symptoms after surgical repair of CHD, 12–14% of children reported new post-traumatic stress disorder symptoms, and greater symptoms were also associated with older age at surgery and less complexity.^33^ Therefore, there may be a need for addressing specific medical fears and post-traumatic stress disorder symptoms in older children with CHD. *Conner’s* measures do not specifically measure medical fears or post-traumatic stress disorder symptoms, but we did find high levels of generalised anxiety disorder symptoms and separation anxiety disorder symptoms, which may be related to medical trauma.

Overall, our findings support the approach that neurodevelopmental follow-up for CHD needs to support all of those at risk and not just target based on course complexity and surgical risk category. Moreover, this study does not explain why commercial insurance was associated with lower anxiety. We speculate that higher anxiety in those without commercial insurance could be related to disparities in care or lack of resources contributing to family stress, but we did not measure family stress or parental anxiety in this study.^26^ Furthermore, increased odds of anxiety symptoms in the lower complexity classification may also be explained by limited sample size (i.e., complete separation), which explains the large confidence interval. As indicated in the Methods section, we used Firth’s method to correct the sample size bias due to unbalanced data. Thus, with limited understanding of the aetiology and predictors of anxiety symptoms in the paediatric population, combined with the deleterious effects of childhood anxiety on outcomes, our findings suggest screening for anxiety symptoms should be routinely performed in young children with CHD.

### Limitations

This study is limited by potential selection bias because patients may be referred and evaluated in our neurodevelopmental programme with concerns about their mental health, behaviour, or school performance, and the reason for referral was not collected in this study. Referral patterns in our programme have changed, and this is unlikely to be a confounder for future studies. Another limitation of our study is that we are unable to explore variables in our study that have been found to be associated with anxiety such as lower birth weight and longer duration of deep hypothermic circulatory arrest.^5,34^ The majority of our sample was Caucasian, which may limit generalisability to a more diverse population with CHD. This distribution reflects the demographic of our state and programme. Finally, our study focused on the presence of anxiety symptoms rather than diagnoses, which may raise the anxiety prevalence estimate. However, observed elevated caregiver-reported anxiety symptoms and the association with functional impairment in adaptive skills suggest that anxiety symptoms were impacting these children regardless of the presence of diagnosis. An area of future research would be a comparison across child and caregiver ratings and those who go on to receive a formal diagnosis of anxiety.

## Conclusions

Anxiety symptoms in elementary-aged children with CHD are common and associated with functional impairments to adaptive skills. Our study suggests that targeted anxiety symptom screening, referral for formal anxiety diagnosis, and early referral for treatment may be a beneficial addition to the standard of care in neuro-developmental follow-up programmes for elementary-aged children with CHD, particularly because of the absence of consistent clinical (e.g., surgical complexity) and personal factors (e.g., age) that may be associated with anxiety. The association of anxiety symptoms with functional impairments in adaptive skills provides evidence that targeted anxiety screening should be a clinical and research priority in developmental follow-up programmes for children with CHD.

## Figures and Tables

**Table 1.** Patient population: clinical and demographic characteristics stratified by age groups. n = 194. Values are frequencies (%) for categorical variables and median (interquartile range,) for continuous variables

	Early elementary	Late elementary
	(n = 78)	(n = 116)
Anxiety symptoms	49 (63)	91 (78)
Global functional impairment	35 (45)	44 (38)
Age at evaluation in years	4.8 (4.3, 6.0)	9.3 (8.2, 11.8)
Sex		
Male	57 (73)	69 (59)
Insurance		
Private	49 (64)	80 (69)
Medicaid	26 (34)	31 (27)
Self-pay	2 (3)	5 (4)
Race		
Caucasian	60 (77)	103 (89)
Other or declined to answer	18 (23)	13 (11)
Family income		
$30,000–50,000	5 (6)	5 (4)
$50,000–70,000	55 (71)	89 (77)
$70,000–90,000	18 (23)	22 (19)
Cardiac anatomy		
Single ventricle	24 (31)	53 (46)
Two ventricle	54 (69)	63 (54)
Prenatal diagnosis		
Yes	35 (45)	53 (46)
No	39 (50)	58 (50)
Unknown	4 (5)	5 (4)
RACHS		
Low complexity	13 (17)	19 (16)
Medium complexity	40 (51)	53 (46)
High complexity	21 (27)	39 (34)
Scoring not applicable (no surgery)	4 (5)	5 (4)
Index surgery hospitalisation length (days)	16.5 (10, 28.5)	14.5 (7.0, 27.2)
Age at first operation (days)	10.0 (6.8, 63.0)	8.5 (5.0, 34.5)
Complications		
Yes	50 (64)	74 (64)
No	28 (36)	42 (36)
Number of medications at follow-up		
0	24 (32)	28 (24)
1–3	40 (53)	63 (54)
4+	12 (15)	25 (22)

Abbreviations: RACHS – risk adjustment for congenital heart surgery.

**Table 2.** Univariate analysis of association of demographic and clinical factors with anxiety symptoms in each age group

	Early elementary		Late elementary	
Variables	OR (95% CI)	p-value	OR (95% CI)	p-value
Age at evaluation (years)	0.93 (0.6, 1.5)	0.75	1.03 (0.8, 1.3)	0.80
Sex:	0.8 (0.3, 2.2)	0.67	0.63 (0.2, 1.6)	0.33
Male				
Female	–	–	–	–
Insurance:				
Private	0.25 (0.1, 0.7)	0.014*	1.67 (0.7, 4.2)	0.28
Public or self-pay	–	–	–	–
Race:				
White or Caucasian	0.8 (0.3, 2.4)	0.70	1.1 (0.2, 4.0)	0.89
Other	–	–	–	–
Family income by county	1.24 (0.4, 4.0)	0.70	3.24 (0.9, 21.3)	0.13
Cardiac anatomy:				
Two ventricle	1.69 (0.6,4.6)	0.29	0.88 (0.4, 2.1)	0.77
Single ventricle	–	–	–	–
Prenatal diagnosis:				
Yes	0.51 (0.2, 1.3)	0.16	0.72 (0.3, 1.8)	0.48
No or unknown	–	–	–	–
RACHS:				
Low complexity or not applicable	1.45 (0.36, 6.24)	0.60	12.15 (1.4, 1598.89)	0.019*
Medium complexity	2.12 (0.71, 6.42)	0.18	0.71 (0.27, 1.77)	0.46
High complexity	–	–	–	–
Index surgery hospitalisation length (days)	1.01 (0.9, 1.03)	0.56	1.01 (1.01, 1.03)	0.51
Age at first operation (days)	1 (1.0, 1.0)	0.93	1.01 (1.0, 1.02)	0.17
Number of medications:				
4+	1.8 (0.4, 9.7)	0.46	2 (0.5, 10.4)	0.37
1–3	0.9 (0.3, 2.5)	0.84	0.8 (0.3, 2.3)	0.68
0	–	–	–	–
Complications:				
Yes	1 (0.4, 2.7)	>0.99	0.79 (0.3, 2.0)	0.62
No	–	–	–	–

Abbreviations: RACHS – risk adjustment for congenital heart surgery.

* p-value < 0.05.
